# Research on the impact of artificial intelligence on EFL learners’ foreign language anxiety

**DOI:** 10.3389/fpsyg.2026.1847269

**Published:** 2026-08-26

**Authors:** Shiyu Wang, Ying Cui, Xiaoquan Pan

**Affiliations:** 1 College of Foreign Languages, Zhejiang Normal University, Jinhua, China; 2 Xingzhi College, Zhejiang Normal University, Jinhua, China; 3 College of Foreign Languages and International Education, Shanghai Jian Qiao University, Shanghai, China

**Keywords:** affective factors, artificial intelligence, English as a foreign language (EFL), foreign language anxiety, systematic review

## Abstract

**Introduction:**

As learners of English as a Foreign Language (EFL) frequently experience foreign language anxiety (FLA), a significant negative affective factor that adversely impacts learning outcomes warrants scholarly attention. Recent trends show an increasing integration of artificial intelligence (AI) in language education to prompt investigation into AI’s effectiveness on EFL learners’ foreign language anxiety. Nevertheless, systematic reviews synthesizing empirical findings on the effects of AI’s applications on EFL learners’ FLA remain scarce.

**Methods:**

This study employed the PRISMA framework to conduct a comprehensive review of peer-reviewed empirical research published between January 2016 and December 2025.

**Results:**

Based on systematic analysis, the findings are categorized into four dimensions: general characteristics of the researches, AI affordances and effectiveness, possible mechanism, other contributing factors. In addition, the factors influencing AI’s impact on learners’ FLA can be primarily synthesized into two sides, internal factors (learner characteristics, perceptions) and external factors (subjective elements and objective elements).

**Discussion:**

This review thereby elucidates possible potential pathways through which AI influences EFL learners’ foreign language anxiety. Finally, the implications highlight the need for careful consideration of factors affecting AI implementation efficacy in language education. However, the proposed possible mechanism and influencing factors remain provisional and still require empirical testing in future studies.

## 1 Introduction

The process of learning English as a Foreign Language (EFL) frequently presents cognitive demands and evokes negative emotional states. Among these, Foreign Language Anxiety (FLA) constitutes a critically influential emotional variable in language acquisition, immensely impacting learners’ outcomes (Pan and Zhang, 2023). Concurrently, the ongoing maturation of Artificial Intelligence (AI) technology has increasingly drawn scholarly attention toward its potential applications within educational contexts. Consequently, there is burgeoning exploration into leveraging AI tools to enhance teaching and learning practices specifically within EFL education. However, extant research investigating the application of AI to mitigate foreign language anxiety among EFL learners remains fragmented, hindering the formation of a comprehensive understanding of this evolving field. This study undertakes a systematic review of research concerning the application of AI interventions to address FLA among EFL learners, with the primary objective of synthesizing current findings and offering guidance for future research and pedagogical practice.

### 1.1 Foreign language anxiety

Horwitz et al. (1986) defined FLA as “a distinct complex of self-perceptions, beliefs, feelings, and behaviors related to classroom language learning arising from the uniqueness of the language learning process” (p. 128). Scovel (2001) notably asserted that FLA constitutes one of the most significant factors influencing foreign language learning, surpassed only by learning motivation. To elucidate this phenomenon among EFL learners, Horwitz et al. (1986) proposed a seminal FLA model integrating its three primary sources: communicative anxiety (CA), test anxiety (TA), and fear of negative evaluation (FNE). CA manifests as apprehension triggered by actual or anticipated communication, specifically encompassing difficulties in oral expression and comprehension, intrinsically linked to spoken interaction (Horwitz et al., 1986). TA refers to apprehension concerning failure in academic evaluations and examinations. Finally, FNE involves the fear of negative social evaluation—learners’ concern regarding others’ perceptions of their language competence (Dikmen, 2021). This anxiety commonly stems from learners’ psychological need to make a favorable impression in social situations, compounded by worries about appearing incompetent (Teimouri et al., 2019). Consequently, Horwitz et al. (1986) developed the Foreign Language Classroom Anxiety Scale (FLCAS) to measure learners’ anxiety levels during foreign language study.

As a complex linguistic experience, foreign language learning intrinsically involves FLA, which can either impede or facilitate the learning process (Alpert and Haber, 1960; Kleinmann, 1977; Young, 1991; He, 2018). Therefore, investigating the impact of FLA within EFL contexts holds considerable significance. A substantial body of literature demonstrates that FLA exerts a detrimental effect on both the learning process and resulting outcomes. Research indicates that high levels of FLA represent a major obstacle to successful language acquisition (MacIntyre and Gardner, 1994; Liu, 2019; Yim, 2014). For instance, FLA can impair learners’ oral performance and academic achievement, prompting reticence, avoidance behaviors, the development of poor study habits, and potentially even physical symptoms of distress (Horwitz, 2010, 2017; MacIntyre, 2017). Avoidance behavior, specifically, entails learners’ hesitation or unwillingness to actively participate by answering questions, engaging in discussions, or using the target language, thereby depriving them of crucial developmental practice and feedback opportunities (Gregersen and Horwitz, 2002; Woodrow, 2006). Furthermore, anxiety can obstruct cognitive processing (MacIntyre and Gardner, 1994) and diminish learning motivation (Teimouri et al., 2019). It consumes cognitive resources within working memory, constraining the capacity available for routine tasks; consequently, anxiety-related cognitive activity hinders information processing and overall performance (Eysenck, 1979; He, 2018). Such anxiety-induced cognitive interference impairs learners’ ability to process complex linguistic input and retrieve prior knowledge (MacIntyre and Gardner, 1994; MacIntyre, 1995; Chen and Chang, 2009; Jiang and Dewaele, 2019). Ultimately, persistent FLA detrimentally affects learners’ motivation and self-confidence, ultimately undermining their communicative competence and overall language proficiency (Liu and Jackson, 2008; Teimouri et al., 2019).

FLA manifests in various modality-specific forms. Foreign Language Speaking Anxiety (FLSA), representing a specific manifestation of FLA, denotes the anxiety and apprehension learners experience when communicating using a second language in a non-native environment (Woodrow, 2006; Mora et al., 2024). Unlike general FLA, FLSA specifically pertains to spoken interaction contexts. Regarded as a primary impediment to successful language learning (Zhao, 2024), numerous meta-analyses confirm its adverse effects on learner performance and overall proficiency (MacIntyre, 2017). Research indicates that FLSA is the most salient factor influencing learners’ willingness to communicate (Dewaele and Dewaele, 2018). It hinders learners’ capacity for spontaneous expression, reduces the quality of oral responses, and diminishes engagement in language interactions, thereby impeding progress in oral language skills (MacIntyre and Gregersen, 2022). The causes of FLSA are multifaceted, encompassing internal (learner-related) factors—such as fear of making mistakes (Ebadi and Azizimajd, 2024) or self-doubt regarding speaking abilities (Hong and Tai, 2025)—and external factors—including limited daily practice opportunities (Richards, 2015), restricted classroom time (Suzuki and Hanzawa, 2022), fear of negative evaluation from peers and instructors, and a competitive or unsupportive classroom climate (Zheng and Guo, 2024).

Foreign Language Reading Anxiety (FLRA), distinct from general FLA, constitutes a specific form of anxiety affecting learners’ reading comprehension in the target language (Saito et al., 1999). Anxious second-language readers may struggle with unfamiliar vocabulary, grammar, and cultural contexts while also finding it difficult to grasp the overall meaning of texts (Saito et al., 1999). It is noteworthy that FLRA negatively impacts learners on both cognitive and conative levels. Cognitively, FLRA exhibits a negative correlation with reading performance; learners experiencing higher anxiety levels tend to achieve poorer comprehension outcomes (Saito et al., 1999). A recent meta-analysis on FLRA indicated a moderate negative association with foreign language reading performance (Li, 2022). Conatively, reading anxiety demonstrates a moderate to strong negative correlation with reading self-efficacy (Li, 2022).

Foreign Language Listening Anxiety (FLLA) represents another significant form of FLA, typically arising from the cognitive demands inherent in processing auditory input in an unfamiliar language (Vogely, 1999). This anxiety impedes both listening comprehension and overall language acquisition. Contributing factors to heightened listening anxiety levels include the fear of misunderstanding or inability to keep pace with auditory input, low self-esteem and feelings of inadequacy, as well as the inherent unfamiliarity and ambiguity of certain sounds and speech patterns in the target language (Dunkel, 1991; Horwitz et al., 1986; MacIntyre, 1995; Vogely, 1998, 1999). The detrimental impact of listening anxiety on performance is well-established: studies consistently reveal a pronounced negative correlation between listening anxiety and listening proficiency, demonstrating how heightened anxiety hinders auditory comprehension (Zhang, 2013; Kimura, 2017; Zhang, 2019).

Furthermore, Foreign Language Writing Anxiety (FLWA) is defined by the emotional, cognitive, and behavioral characteristics present during the initiation, continuation, and completion of assigned writing tasks in the target language (Bloom, 1985). The theoretical underpinnings trace back to Daly and Miller (1975) who first conceptualized writing anxiety as “writing apprehension,” referring to an individual’s propensity to either accept or avoid writing tasks under specific circumstances or within particular topics during the writing process, developing the Writing Apprehension Test (WAT) for native speakers. Cheng et al. (1999) adapted the WAT to investigate FLWA among students in Taiwan, China. Their findings suggested that FLWA is distinctive from general writing anxiety; it constitutes a specific type of learning anxiety associated uniquely with the process of producing written output in a foreign language. Consequently, scholars have pursued in-depth investigations into its etiology. For English majors, FLWA typically originates from deficiencies in linguistic knowledge, instructors’ excessive demands coupled with insufficient writing guidance, fear of negative feedback, unsatisfactory writing grades, and perceived time constraints (Olanezhad, 2015; Miri and Joia, 2018). Conversely, for non-English majors, FLWA often stems from a lack of writing skills (e.g., weak linguistic foundations, insufficient vocabulary), limited exposure to English, fear of negative evaluation, unrealistically high expectations, insufficient writing practice, and slow composing speed (Liu and Ni, 2015; Rezaei and Jafari, 2014; Kusumaningputri et al., 2018). Consequently, these findings suggest partial overlap in the primary sources of FLWA between these two learner groups.

### 1.2 AI Application in FLA

Driven by the ongoing advancement and refinement of Artificial Intelligence (AI) technology, an expanding range of novel AI applications is being integrated into the field of EFL education. Research examining the application of AI to alleviate foreign language anxiety has primarily focused on two key aspects: (1) changes in the severity of overall foreign language anxiety or its specific manifestations, and (2) changes in anxiety-related variables and correlates.

First, researchers have explored AI’s impact on the overall level of FLA. For instance, Tai and Chen (2020) implemented a 2-week intervention study using Google Assistant with adolescent EFL learners, demonstrating its effectiveness in reducing students’ speaking anxiety. Similarly, El Shazly (2021) employed AI within conversational scenarios to assess its effectiveness on learners’ speaking skills and anxiety levels, observing a significant decrease in anxiety. Furthermore, the private, self-directed learning environments facilitated by AI technology can help mitigate anxiety arising from low self-esteem and apprehension about negative evaluations, which consistently aligns with established findings on antecedents of listening anxiety (Horwitz et al., 1986; Dunkel, 1991).

Second, researchers have investigated the potential impact of AI on foreign language anxiety by examining changes in associated correlates and constructs. For example, Zhang et al. (2024) evaluated the effects of an AI-powered speaking program on EFL learners’ foreign language enjoyment (FLE), foreign language anxiety (FLA), and willingness to communicate (WTC). Their findings revealed that the program extensively enhanced learners’ FLE and WTC while effectively reducing FLA levels. Dizon and Gold (2023) filled a gap in previous studies, that is, AI’s influence on affective factors, discovering that the AWE tool can effectively alleviate learners’ writing anxiety. Çakmak (2022) conducted a 12-week intervention study utilizing Replika (an AI chatbot) with EFL participants. Results indicated that while the chatbot intervention improved speaking performance, it did not necessarily lead to a reduction in speaking anxiety. This suggests a complex relationship among interactions with AI agents, speaking proficiency, and anxiety, indicating that interaction *per se* may not be the sole determinant of anxiety reduction and implying the possible role of mediating or moderating factors.

### 1.3 Existing reviews

The proliferation of AI-driven chatbots for oral language practice has prompted significant research activity. Du and Daniel (2024) conducted a systematic review within this domain, affirming the numerous advantages these tools offer for spoken language training. Their review confirmed that while chatbots can effectively mitigate anxiety and enhance spoken language skills, further investigation across diverse educational contexts is needed to validate their broader applicability and impact. However, Du and Daniel (2024) did not systematically categorize different types of AI tools or examine the consistency of their anxiety-reducing effects across varying learner demographics. Similarly, Fathi et al. (2024) synthesized research on AI tool types used in EFL speaking instruction and evaluated their efficacy for improving learners’ speaking abilities. Their findings indicated that these tools enhance speaking proficiency by promoting fluency, alleviating anxiety, and fostering learner confidence.

Current review literature on AI applications within EFL education predominantly focuses on its role in enhancing general language proficiency or specific practice skills, synthesizing evidence related to improving discrete English competencies (e.g., speaking, writing, vocabulary). However, systematic investigations specifically exploring AI’s impact on negative affective variables among EFL learners, such as foreign language anxiety (FLA)—particularly the factors associated with FLA, the mechanisms through which these factors operate, or the specific pathways by which AI might exert influences on learners—remain scarce. Consequently, despite the growing interest in applying AI within EFL contexts, a critical gap persists: the lack of a systematic literature review synthesizing existing empirical findings on the impact of AI on EFL learners’ FLA and offering substantive recommendations for future research directions in this specific domain.

Given the detrimental effects of FLA on EFL learning processes and the concurrent expansion of research on AI in EFL education, a comprehensive synthesis of this intersecting field is imperative. First, providing researchers and practitioners with an overview necessitates examining the foundational characteristics (e.g., publication trends, geographical focus, educational levels, participant demographics) of the existing literature. Second, as extant empirical studies examine AI’s impact on EFL learners’ FLA from multifaceted perspectives—encompassing behavioral, cognitive, and affective dimensions—a synthesis of this evidence is essential to develop a coherent understanding of AI’s holistic effects. Third, because the mechanisms through which AI influences EFL learners’ FLA are potentially multifaceted and complex, establishing a possible analytical framework to examine possible mechanism is warranted. Fourth, considering that numerous studies have identified factors influencing both the deployment and effectiveness of AI interventions and their subsequent impact on learners, it is necessary to systematically identify potential mediating and moderating factors across key dimensions (e.g., learner characteristics, AI design features, implementation context, pedagogical integration, teacher support). Such an analysis will enhance understanding for both researchers designing future studies and practitioners implementing AI solutions.

### 1.4 Current study

To address these research gaps, the current study therefore aims to conduct a systematic review investigating the impact of AI technologies on foreign language anxiety among EFL learners. Specifically, this review seeks to answer the following research questions (RQs) outlined in Table 1:

**TABLE 1:** Research questions.

Research questions (RQs)	Focus
RQ1: What are the general characteristics of the studies on AI in EFL learners’ FLA?	Publication year, country or region, education level, learning subject, study design;
RQ2: Why can AI exert influences on EFL learners’ FLA?	AI affordances and effectiveness on EFL learners;
RQ3: How does AI influence EFL learners’ FLA?	The possible mechanism of AI towards FLA;
RQ4: What factors may affect the impacts of AI on EFL learners’ FLA?	Factors influencing the application or effects of AI in EFL learners’ FLA

Addressing RQ1: We synthesized the basic characteristics (e.g., publication year, geographical context, educational level, participant profiles, study design) of the selected empirical studies.

Addressing RQ2: We consolidated the documented affordances and effects of AI interventions on EFL learners’ learning outcomes.

Addressing RQ3: We developed a two-pathway framework (direct, indirect) to analyze the potential mechanism through which AI might be instrument in EFL learners’ levels of FLA.

Addressing RQ4: We identified and synthesized salient internal and external factors that may affect the efficacy and outcomes of applying AI to address FLA, focusing on dimensions such as learner perceptions, technological limitations, pedagogical implementation.

## 2 Methods

This study employed a systematic review methodology, primarily conducting qualitative content analysis of the selected published researches to collect and analyze data on the impact of AI on EFL learners’ FLA, covering publications from January 2016 to December 2025. To ensure transparency in the data collection process, this study adhered to the Preferred Reporting Items for Systematic Reviews and Meta-Analyses (PRISMA) framework, systematically extracting and analyzing general information, including author details, country or region, year of publication, measurement tools, participants, and results.

### 2.1 Literature research

To collect literature, we searched three major databases: Web of Science (WOS), Scopus, and ProQuest. The search spanned from January 2016 to December 2025, focusing chiefly on the impact of AI on EFL learners’ FLA. The three main keywords were “artificial intelligence,” “English as a foreign language learners,” and “foreign language anxiety,” supplemented by abbreviations such as AI, generative AI, GenAI, EFL learners, EFL, and FLA. To ensure a more comprehensive search, additional keywords including “artificial intelligence,” “AI-driven,” “AI-powered,” “AI-assisted,” “English as a foreign language,” “language anxiety,” and “anxiety” were also included. Boolean operators (such as AND and OR) were used to link these terms, thereby creating a search strategy that ensures comprehensive coverage of relevant literature while maintaining subject specificity. The search query is set as follows: ((“artificial intelligence” OR “AI” OR “AI-driven” OR “AI-powered” OR “AI-assisted” OR “generative AI” OR “GenAI”) AND (“EFL” OR “English as a foreign language” OR “English as a foreign language learners” OR “EFL learners”) AND (“language anxiety” OR “foreign language anxiety” OR “FLA” OR “anxiety”)). This search strategy was applied consistently across all these three databases’ advanced search except for the beginning (WOS: TS = , Scopus: TITLE-ABS-KEY, ProQuest: noft). This search yielded a total of 264 records.

### 2.2 Inclusion and exclusion criteria

To ensure the relevance and quality of these articles, a set of inclusion and exclusion criteria was applied to select them. Studies must focus on the impact of AI on EFL learners’ FLA and must be original empirical research articles. Given that the search keywords included “EFL” and most studies were published in English, the language was restricted to English. Additionally, the document type was limited to articles published in peer-reviewed academic journals, excluding conference proceedings, reviews, and book chapters. Accordingly, studies were excluded if any of the following conditions applied: (1) the full text was unavailable or lacked basic structural elements (such as introduction, methodology, results, and conclusion); (2) duplicate entries were present (screened using Zotero 7.0.8’s automatic deduplication feature and supplemented by manual screening); (3) studies that did not focus on foreign language anxiety, such as those dealing solely with general anxiety, were outside the scope of this study.

### 2.3 Article screening and selection process

A total of 264 initial records were retrieved from the three databases (WOS: 80; Scopus: 125; ProQuest: 59). After deduplication, 170 studies remained and were imported into Zotero for further screening. In the first stage, one non-English article was excluded, and 31 other types of articles (such as conference proceedings, systematic reviews, and book chapters) were removed. In the second stage, screening was initially conducted based on article titles and abstracts with the efforts of two researchers’ independent evaluation. 105 articles that had no or only a weak association with the search keywords, were included in the screening scope because the search terms appeared in their abstracts or titles. Then the remaining 33 articles underwent a full-text review, in this phase, 1 article did not focus on AI-driven learning environments (e.g., it was set in a conventional classroom setting); 8 papers did not focus on foreign language anxiety (e.g., they merely examined learners’ psychological anxiety or changes in anxiety levels interfered by other factors), which did not align with the research topic. After excluding the aforementioned papers, the authors of this study reviewed the selected articles, and 1 paper was eliminated due to the unavailability of the full text. To ensure the quality of the sources, this review defines “core journals” as SSCI publications; non-SSCI publications will be excluded. Thus, 2 papers from non-core journals were also excluded. After all disagreement were resolved, ultimately, 21 empirical research papers that met the requirements and were both relevant and representative were selected.

The methodological quality of these included studies was evaluated by using the Mixed Methods Appraisal Tool (Hong et al., 2018). This tool can be allowed to appraise the methodological quality of qualitative research, randomized controlled trials, non-randomized studies, quantitative descriptive studies, and mixed methods studies. Each included study was independently assessed by two reviewers based on MMAT criteria. All the disagreements were resolved during the discussion.

### 2.4 Analysis and coding process

**FIGURE 1:**
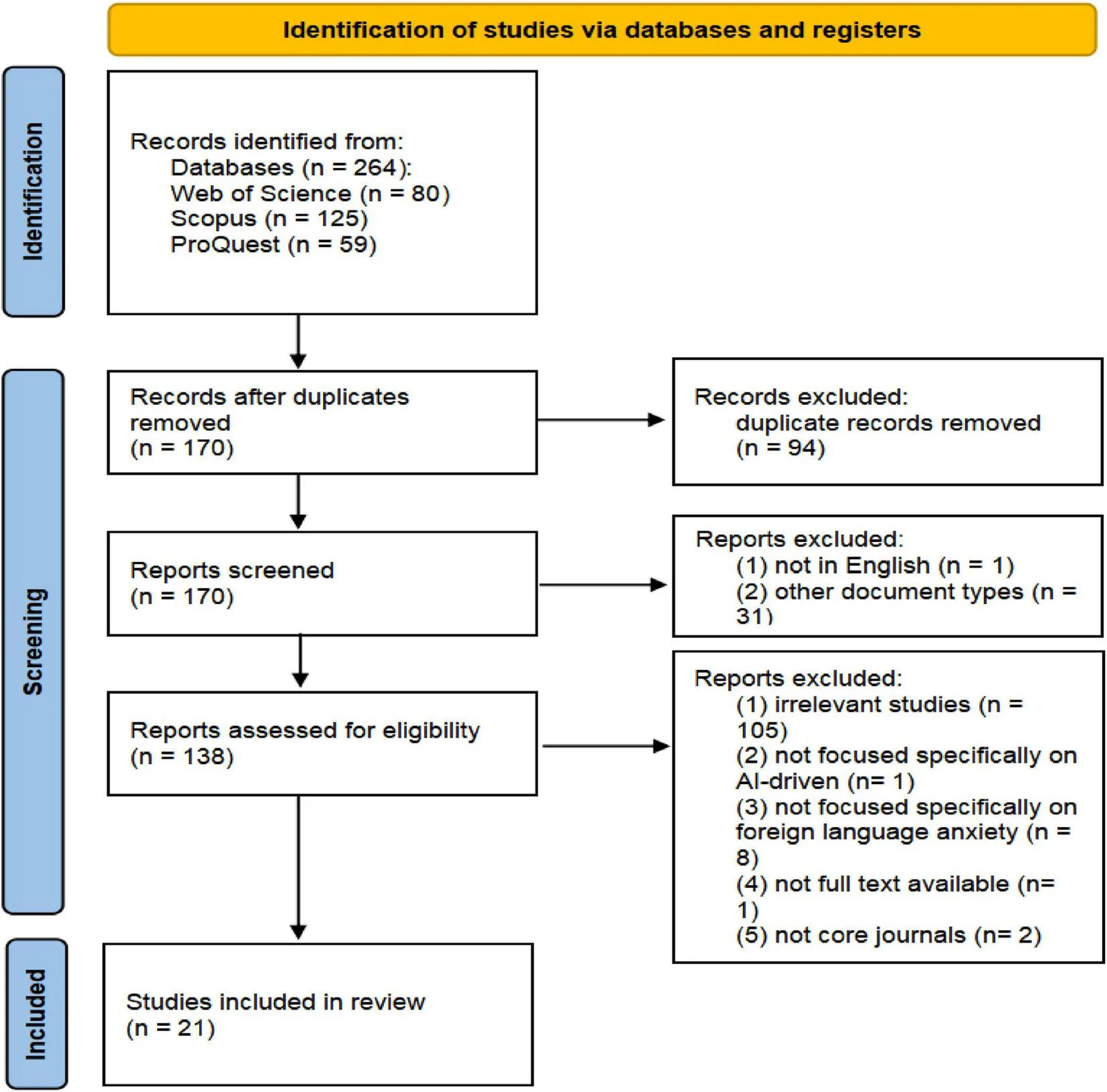
The PRISMA flow chart for literature search and selection process.

Details of the 21 studies ultimately included in the final analysis are presented in the PRISMA flow diagram (Figure 1). Basic information from the selected literature was extracted and categorized using an Excel worksheet. In the initial phase, general information—including publication year, country or region, study design, study setting, learning subject, and participants’ educational background—was extracted. During the in-depth analysis phase, content analysis was employed to synthesize data based on three core characteristics of each study: (1) the affordance of each type of AI technology on EFL learners’ FLA; (2) effectiveness of AI on EFL learners’ FLA and learning outcomes; and (3) other contributing factors. Finally, overall research trends and the unique features of each study will be presented in Supplementary Appendix 1. The details of these elements and the rationale behind the coding process are elaborated as follows.

The “author (year)” column is recorded in the format of the first author’s surname and the publication year, representing a unique identity of each study. The code for “country or region” refers to where the study was conducted, or the country or region of the authors or first author if the relevant information about the study location was not provided. The methods described in the “study design” section are categorized as quantitative, qualitative, and mixed-methods approaches, which helps to understand the research rigor design trends. The code for “participants’ educational background” include elementary, junior secondary, senior secondary and tertiary to help understand learner type and education level. The “learning subjects” in EFL include speaking, reading, writing, listening, and general English skills if not specifically focusing on one skill. The “study setting” code is classified into formal learning and informal learning to reflect the different contexts in which AI technologies affecting on FLA.

To understand the possible mechanism and role of these AI tools, these tools are categorized into different types based on their core technological function and application context. The “affordance of AI technology” is determined according to study’s introduction of the used AI in “literature review” section and learners’ perceptions or attitudes in “results/findings” section. The “effectiveness of AI on EFL learners FLA” is recorded based on the changes of participants’ FLA level in the “results/data collection and analysis” section, and the “effectiveness of AI on EFL learners learning outcomes” are determined by reviewing the statements described in “results/data collection and analysis” section on the basis of CAC model, which is classified into cognitive, affective, and conative. “Other contributing factors” are based on participants’ interview and perception in “results/findings” part, which can be categorized into participant-related (internal) and contextual (external). Two coders were involved in coding process and analyzed independently. For any coding discrepancies, the two coders presented their respective reasons and then discussed them; if they could not reach an agreement, the third author made the final decision. All disagreements were resolved after further discussion.

## 3 Results

### 3.1 What are the general characteristics of studies on AI in EFL learners’ FLA?

**FIGURE 2:**
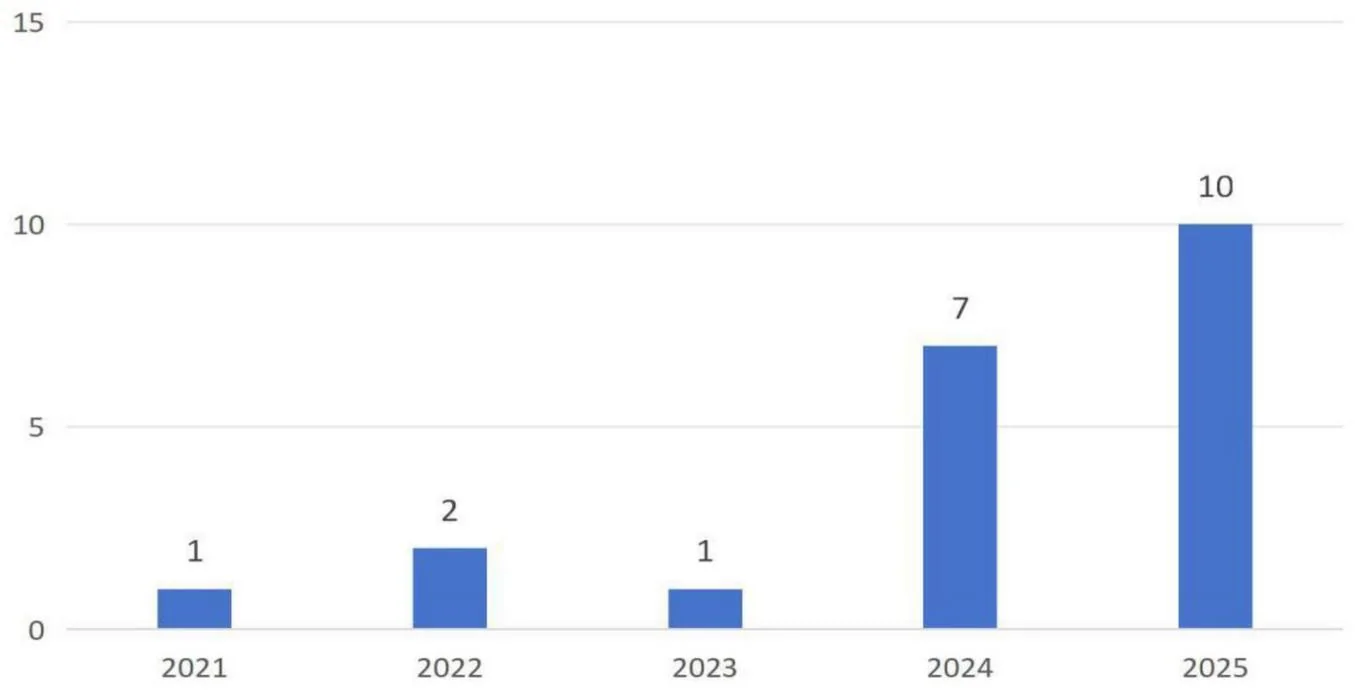
Number of studies across years.

**FIGURE 3:**
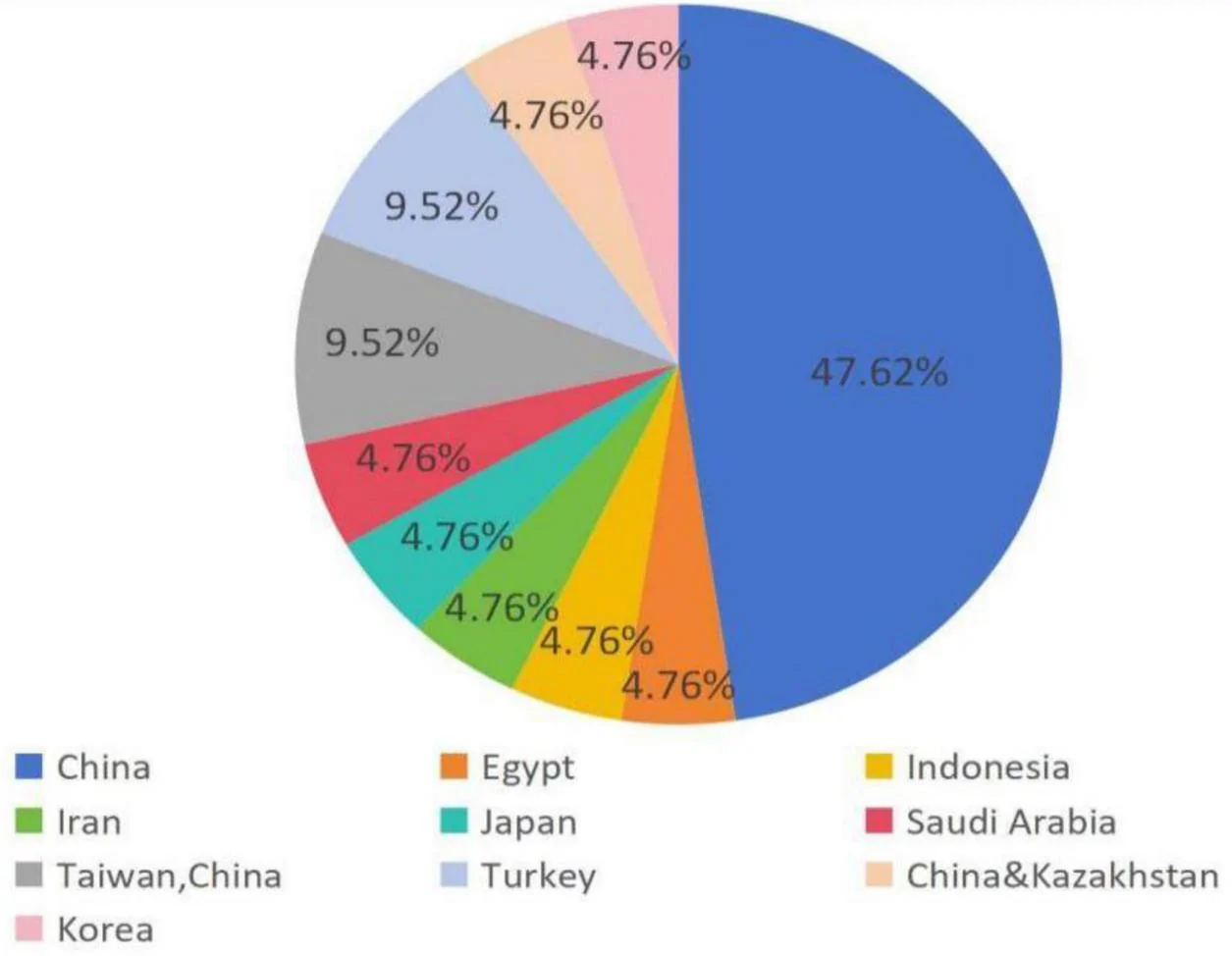
Proportion of studies conducted in countries or regions.

**FIGURE 4:**
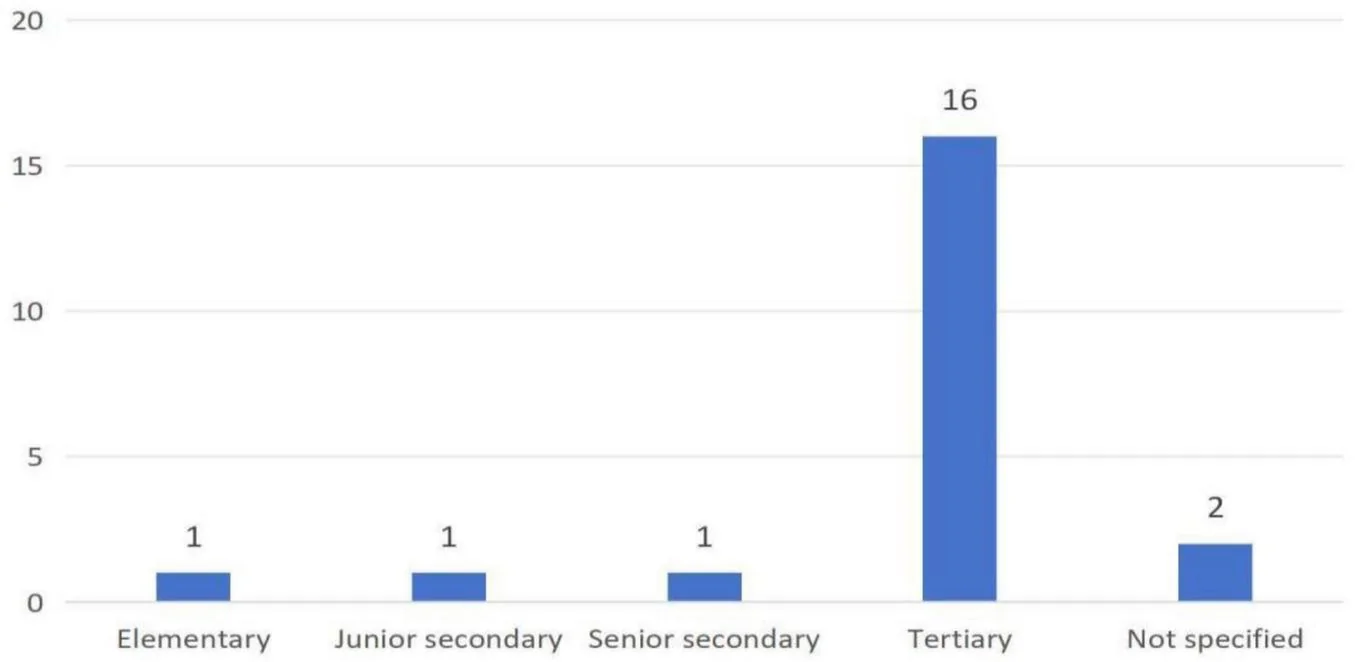
Numbers of studies at different education levels.

**FIGURE 5:**
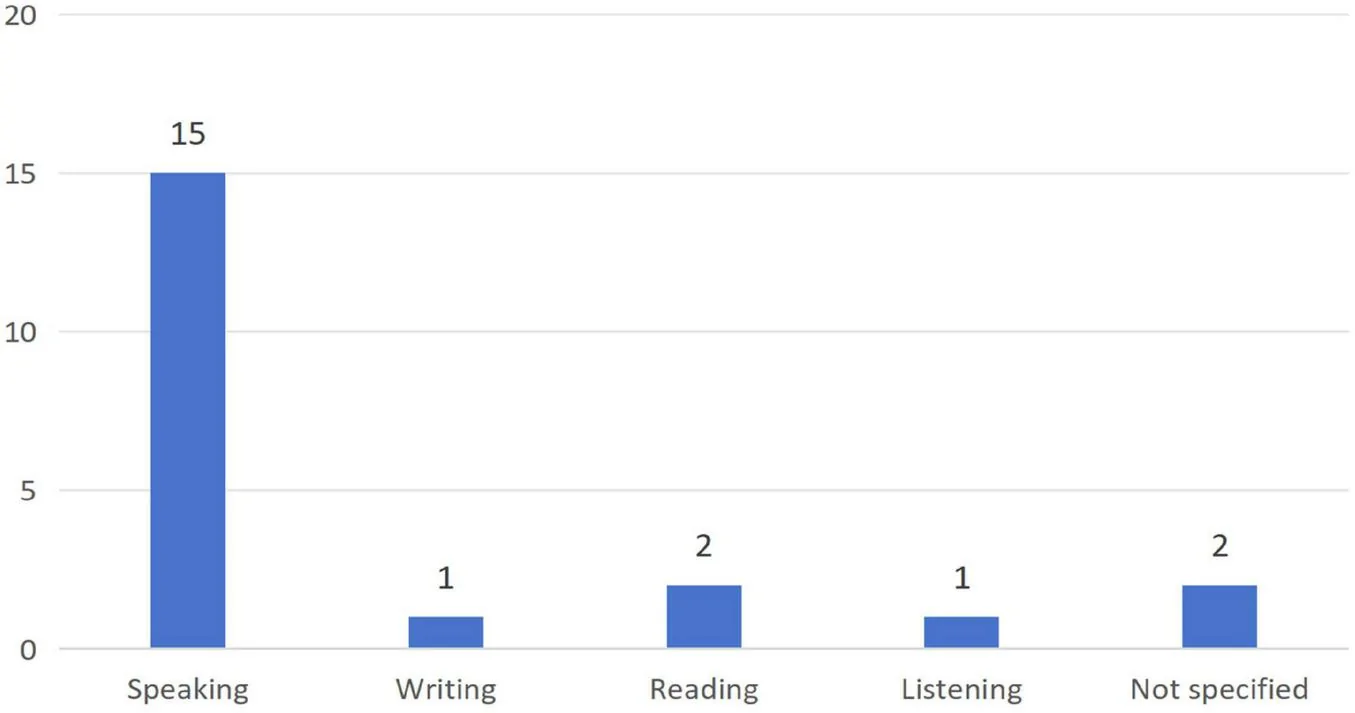
Numbers of studies focusing on different learning subjects.

(1) Publication year. The included publications spanned from May 2021 to November 2025. Figure 2 illustrates that the majority of selected articles were published in 2024 and 2025, which indicating that there is a growing research interest in foreign language learning emotions. This review indicates that AI applications in EFL education have garnered substantial scholarly attention in recent years, particularly since 2024. The inaugural empirical study examining EFL learners’ Foreign Language Anxiety (FLA) within an AI-enabled context emerged in 2021, investigating the role of AI tools in managing undergraduates’ speaking anxiety during EFL courses. Publication trends (Figure 2) reveal that 2021-2023 constituted an exploratory phase for this research domain. However, as AI technologies advance, publications surged post-2023, reflecting growing recognition that FLA research must account for contemporary technological contexts while evidencing AI’s expanding integration. Scholars now increasingly explore how automatic feedback systems and generative AI impact EFL learners’ FLA. For example, Shadiev et al. (2024) demonstrated speech-enabled corrective feedback (SECF) technology’s efficacy in enhancing speaking skills and reducing FLA, while Choi (2025) found that generative AI not only enhances individual achievement but also cultivates learning communities.

(2) Country or region. As depicted in Figure 3, China constituted the predominant geographic focus, accounting for nearly half (47.62%) of the 21 studies meeting inclusion criteria. Turkey and Taiwan, China followed in frequency, markedly outnumbering contributions from other countries and regions. Geographically, extant research predominantly originates from Asian and Middle Eastern nations. This distribution signals these regions’ pressing need to develop L2 communication competencies for global engagement, coupled with strong receptivity to novel AI applications. This trend aligns with Asia’s accelerating adoption of AI-integrated education, resonates with China’s policy emphasis on educational intelligence, and addresses regional imperatives to elevate English education quality.

(3) Education level and study setting. In terms of study context, according to Supplementary Appendix 1, most researches reveal a tendency to follow with interest the formal learning settings, such as universities or language training institutions (*n* = 19), while only a few studies (*n* = 2) have turned their attention to informal learning. Studies encompassed EFL learners across virtually all educational stages (Figure 4). Research concentration was highest at the tertiary level, accounting for most of the research samples (*n* = 16). Nevertheless, learners of basic education (elementary, junior secondary, senior secondary) are scarcely present, with one study targeting on each education level. Two studies did not specify educational levels: one examined intermediate-level Chinese EFL learners aged 22–32 (Peng, 2025), while the other surveyed students enrolled in IELTS speaking courses at a private language institute (Ebadi et al., 2025). This distribution highlights a conspicuous imbalance in participant selection in research on technology-assisted foreign language learning. It can be speculated that the underlying reason may be that learners at tertiary education level possess stronger self-directed learning competence and a higher degree of adaptability.

(4) Learning subject. Figure 5 demonstrates that speaking skills constituted the primary focus (*n* = 15), followed by writing, listening, and reading skills. Two studies did not specify the language skill examined and were categorized as “not specified.” Concerning focal skills, the literature prioritizes speaking anxiety and speaking skill enhancement, indicative of a scholarly emphasis on leveraging AI chatbots or speech-enabled AI for oral practice. This underscores heightened attention to speaking anxiety and reaffirms speaking as a persistent challenge within EFL acquisition.

(5) Study design. As illustrated in Table 2, the majority of the included studies employed a mixed-methods approach (*n* = 14), while those using solely quantitative methods (*n* = 7) were fewer in number, and no studies utilized qualitative methods. Given the predominance of mixed-methods approaches, it is reasonable to infer that researchers in this field are increasingly recognizing the complexity of AI-assisted language learning’s impact on learners’ FLA; merely relying on single kind of data is insufficient to fully elucidate research findings. Consequently, combining quantitative measures (pre- and post-tests, anxiety scales, language skills and performance) with qualitative analysis (semi-structured interviews, learner perception) has gradually emerged as a research trend in recent years, which reflects a growing emphasis on integrating multidimensional perspectives. Purely quantitative studies are often used to explore AI technologies that show pedagogical potential but have not yet been formally implemented, or the integration of AI technologies with novel teaching models (Li and Long, 2025), AI-assisted simplification of reading texts (Çelik et al., 2024), and the integration of dynamic assessment into speech recognition systems (Chen et al., 2022). such studies tend to gather concentration more on the direct impact of technological tools on EFL learners’ FLA levels and language proficiency. It is worth noting that none of the selected studies adopted qualitative methods, indicating that future research in this field could further explore learners’ perspectives and emotional experiences to address the lack of attention to participants’ agency in quantitative studies.

**TABLE 2:** Numbers of study design.

Study design	Mixed	Quantitative	Qualitative
Number	14	7	0

### 3.2 Why can AI exert influences on EFL learners’ FLA?

**FIGURE 6:**
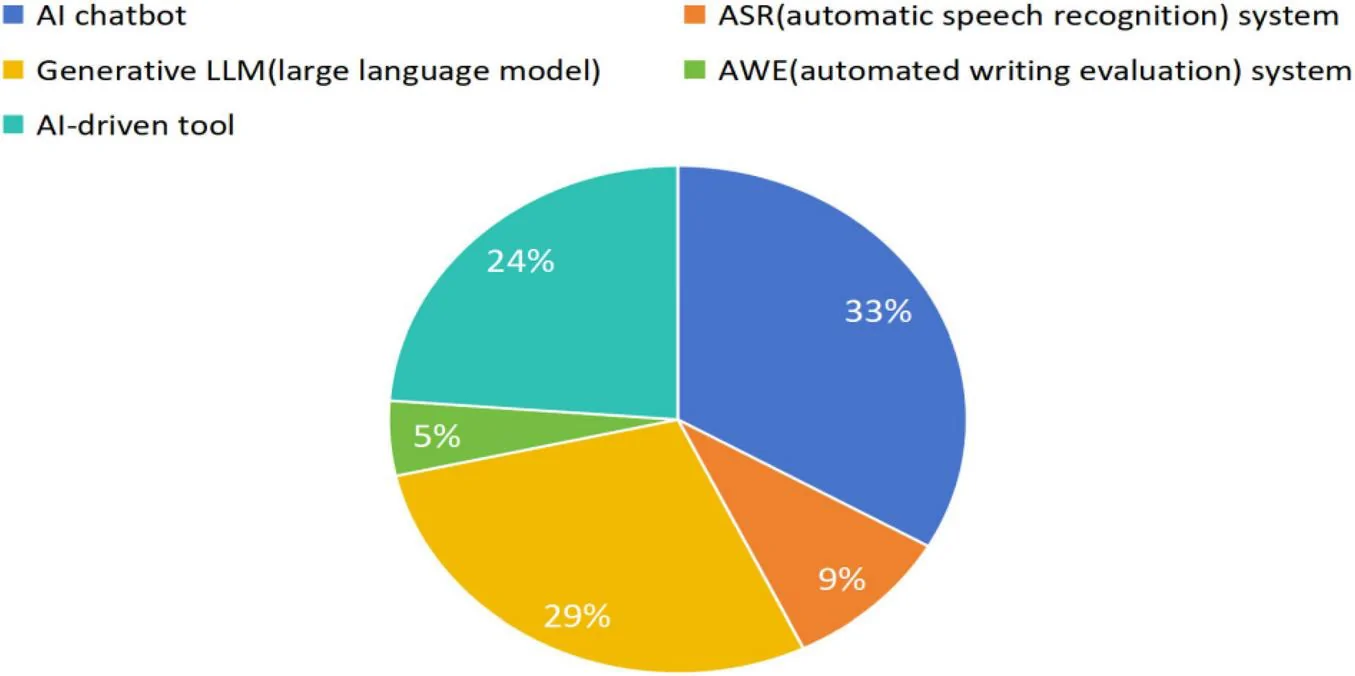
Proportion of AI types used in FLA studies (2021–2025).

**FIGURE 7:**
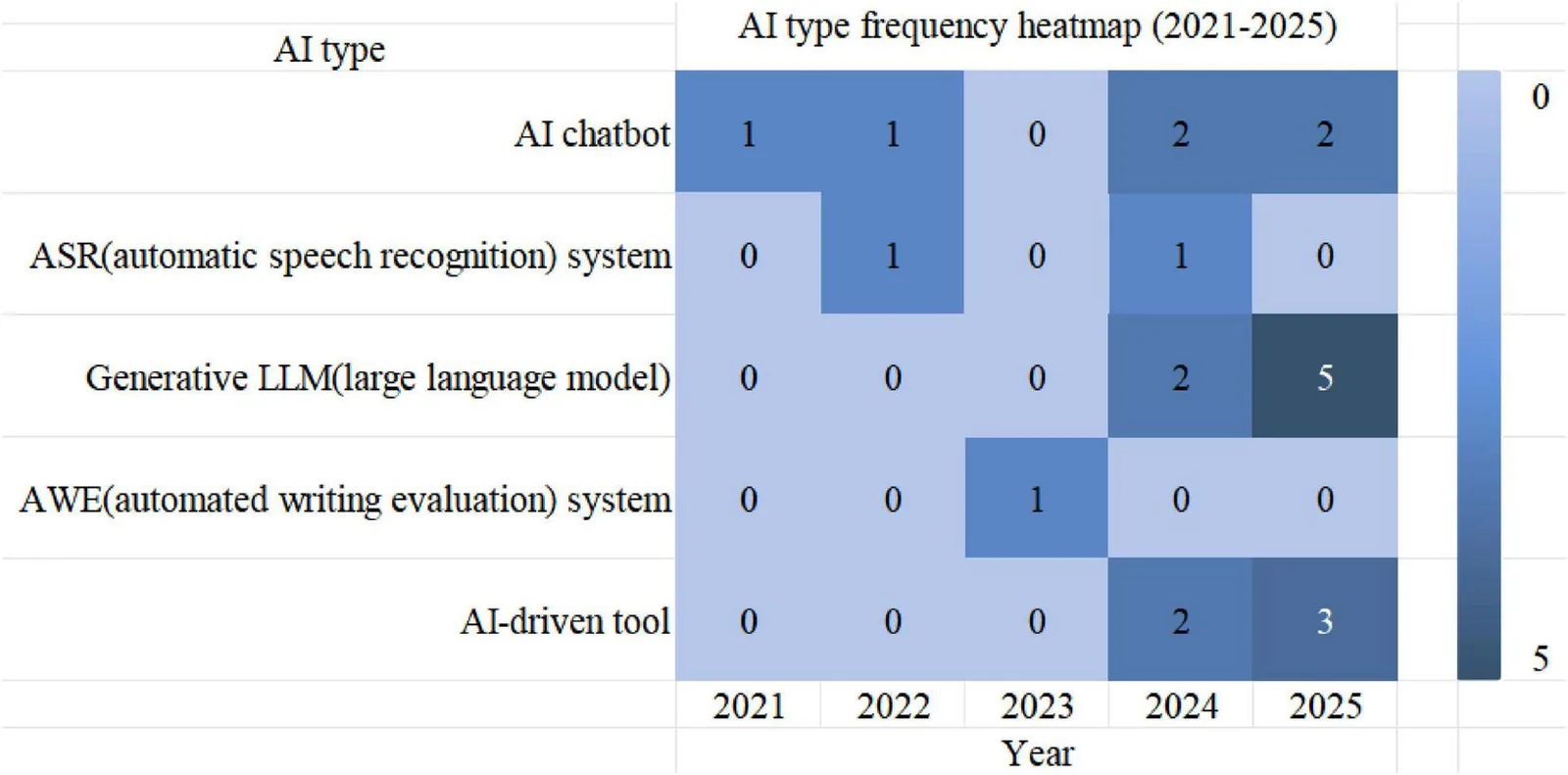
AI types frequency heatmap (2021–2025).

The effectiveness of AI on English as a Foreign Language (EFL) learners’ Foreign Language Anxiety (FLA) varies across different application types. As synthesized from the selected studies, Figures 6, 7 delineate the proportion and tendency of distinct AI categories used in FLA researches recent years.

In this article, based on the primary types of interaction between AI and human according to the descriptions of the basic technical characteristics and titles of the included studies, as well as prior researches in related field, AI is divided into five major categories: AI chatbots, generative large language models (LLMs), automatic speech recognition (ASR) systems, automated writing evaluation (AWE) systems, and AI-driven tools. Therefore, AI chatbots are primarily used for spoken interaction, generative LLMs specialize in written interaction, ASR systems focus on speech recognition with no interaction, and AWE systems is characterized by written text recognition. Since some AI tools incorporate several technical characteristics, they are classified as AI-driven tools.

As depicted in Figures 6, 7, AI chatbots are emerging as a dominant force, accounting for one-third of all categories with their continuous usage in recent years. AI chatbots are gradually becoming an essential tool for second-language learning, providing learners with a convenient platform to practice speaking. By processing commands based on learners’ needs, chatbots can simulate real-life communication scenarios, and create a low-pressure practice environment for learners. In addition, owing to the personalized feedback affordance, chatbots can provide real-time responses and correction suggestions. Particularly in terms of emotional interaction, chatbots can even rival face-to-face communication with peers, alleviating the anxiety associated with the presence of others and thereby boosting self-confidence and the willingness to communicate (Wang et al., 2024; Wu et al., 2025). These factors make chatbots effective tools for second-language speaking practice and development.

At the same time, generative large language models (LLMs), represented by ChatGPT, have unfolded entirely new possibilities for FLA researches. As presented in Figures 6, 7, the proportion is second only to that of AI chatbots, and their usage has been increasing extensively since 2024. Compared to chatbots, LLMs excel in contextual understanding and response generation, which can dynamically adjust based on an individual’s real-time performance and needs, and effectively mitigate learner anxiety and enhance engagement. This more advanced conversational model lays a solid foundation for creating a comfortable environment and an immersive experience. Furthermore, LLMs can design gamified tasks that simultaneously improve language accuracy and fluency while maintaining learner motivation (Peng, 2025).

It is worth noting that AI-driven tools have garnered favor among researchers. Recently, the integration of AI with traditional technologies has broadened research directions, which unveiling novelty and research potential. For example, virtual reality (VR) technology, enhanced by AI, has been used to overcome the fear of public speaking (Chen, 2024). SmallTalk2Me (Ebadi et al., 2025) and Doubao, due to their integrating technologies such as ASR and LLM, have immensely promoted learners’ speaking skills and reduced foreign speaking anxiety, highlighting the advantages of a learner-centered benefits. In addition, the bilingual-adaptive Lora app (Zhang et al., 2024) effectively promotes second-language learning by encouraging learners to engage in supportive conversations and providing both direct and indirect feedback.

However, the application of AI in the field of FLA extends far beyond forementioned AI categories. Automatic speech recognition (ASR) systems and automated writing evaluation (AWE) systems, due to their timeliness and convenience, can reduce unnecessary time consumption and construct a learner-centered learning environment, thereby fostering students’ capacity to become immersed in independent learning.

### 3.3 How does AI influence EFL learners’ FLA?

As indicated in Table 3, the relevant AI effectiveness on EFL learning outcomes identified across the 21 included studies were categorized into three dimensions: cognitive, affective, and conative. This classification draws upon Fishbein and Ajzen’s (1975) Cognition-Affect-Conation (CAC) model, aiming to reveal the intrinsic logical relationship between human consciousness and behavior. The CAC framework, rooted in traditional psychological research, has been refined and applied by numerous scholars over the years. Fishbein and Ajzen (1975) argued that a person’s cognition, affection, and conation are key components in determining one’s attitudes. They emphasized that an individual’s conation, the inclination to exhibit a certain behavior, is governed by their affection toward that behavior, which in turn is shaped by their cognition of it. This theoretical framework illustrates the mechanisms of transformation from perception to action. In this context, cognition encompasses both the perception and understanding of an object; when these two elements combine, they form a belief about that object. Affection refers to an individual’s emotional state or emotional response to cognition. Conation indicates the tendency to take action based on current cognition and affection. In the 21 studies included in this review, the impact of AI on EFL learners’ learning outcomes can be categorized into three types based on this framework, which in turn may explain the possible mechanism through which AI plays a pivotal role in learners’ FLA. Among these three categories of effects, the cognitive effect primarily includes specific language skills and language proficiency, such as speaking skills; the affective effect mainly encompasses FLA and other emotional experiences; and the conative effect refers to motivation, learner autonomy, and WTC. There are two possible pathways of influence: the direct pathway and the indirect pathway.

**TABLE 3:** AI effectiveness on EFL learners learning.

Effects on EFL learners learning outcomes	Cognitive	Affective	Conative
	Language skills, language proficiency	FLA, other emotional experiences	Motivation, learner autonomy, WTC

First is the direct pathway. By fostering a non-judgmental and immersive environment (Zhang et al., 2024; Çakmak, 2022; Chen, 2024; Peng, 2025; Chen et al., 2025), AI allows most EFL learners to avoid the pressure of interacting with peers or teachers and the risk of making mistakes, which may directly reduce learners’ foreign language learning anxiety. Furthermore, some studies have also found an increase in learners’ foreign language enjoyment (FLE) (Zhang et al., 2024; Wu et al., 2025; Peng, 2025; Wang et al., 2024; Choi, 2025). From the perspective of positive psychology (Seligman and Csikszentmihalyi, 2000), by mitigating FLA and boosting FLE, this effectiveness of AI brings a positive learning experience and enhances learning outcomes. Second is the indirect pathway. Another factor that may affect variation in FLA is the cognitive effect. Through repeated AI interventions, learners’ cognitive levels regarding foreign language learning have been elevated tremendously, as evidenced by enhanced language skills or proficiency. Grounded on the CAC model (Fishbein and Ajzen, 1975), affection occupies a central position, serving as a pivotal link between cognition and conation, thus changes in cognition directly lead to changes in affection. According to Bandura’s (1997) theory of self-efficacy, learners in AI-assisted learning environments perceive significant promotion in their capacities through AI-enabled immediate feedback and practice. This direct experience of success boosts self-efficacy, thereby relieving anxiety during subsequent practice sessions. Therefore, AI may indirectly decrease FLA level by influencing EFL learners’ language capacity.

### 3.4 What factors may affect the impact of AI on EFL learners’ FLA?

It is important to acknowledge that several studies have examined learners’ attitudes and perceptions regarding AI and technology-assisted learning tools. Such perceptions highlight their potential role as influential factors in how AI impacts EFL learners’ Foreign Language Anxiety (FLA). These factors can broadly be classified as participant-related (internal) and contextual (external).

First, participant-related factors have a bearing on outcomes. Although most learners presented a positive attitude toward the adoption of AI in language education, some of them still held negative opinions about it. Negative perceptions towards chatbots as conversational partners due to anxious and awkward feelings when interacting with AI, and negative attitudes towards technology use in language learning which attributed to non-essentiality and adverse physical effects like eye strain and diminished attention (Çakmak, 2022; Chen, 2024). Furthermore, research by Ding and Yusof (2025) emphasized the relevance of individual learner differences. Their study, while primarily focused on anxiety levels, noted that factors such as prior language proficiency and digital literacy may also critically shape learners’ interactions with and responses to chatbots. With the comparison between FLA levels of HG(high proficiency group) and LG(low proficiency group), there was a difference in the lifting of the oral proficiency although both groups’ FLA level reduced, which implying that learner’s initial language skill may be a point worth considering (Chen et al., 2025).

Second, contextual factors warrant consideration and can be divided into subjective and objective elements. Subjective elements include evaluations from others and interactions with others. Study findings indicates that respondents’ concerns about teachers continuously correcting their mistakes and their anticipation that classmates might mock their communication performance are both factors that may exacerbate FLA (El Shazly, 2021). Choi (2025) underscored that despite AI’s capability to provide technical feedback, human elements remain crucial in learning. Interactions with peers and teachers were found to substantially affect both learners’ experiences and the effective integration of AI. Moreover, objective contextual factors exert considerable influence. Technical limitations also constitute significant objective factors. As observed in interview results from several studies, some participants expressed that technical issues with the AI itself could affect the user experience and their willingness to continue using it. For instance, unstable internet connectivity can disrupt human-chatbot dialogues, causing response delays; the conciseness, clarity and accuracy of the information should be paid attention to make adjustments during human-chatbot interaction; LLMs have limitations of straying from the topic and being unable to use body language to express participants themselves (Wu et al., 2025; Zheng, 2024; Zheng et al., 2025). While this specific issue did not exert a major observed influence in their investigation, it remains a critical technical consideration for the implementation of AI technologies in language learning contexts.

As shown in Table 4, a subtle phenomenon was observed: the results of three studies indicated that FLA levels did not decrease after AI intervention; instead, they showed a slight increase or no significant change. Considering the intervention duration listed in Supplementary Appendix 1, the studies reporting increased FLA levels were the third and fifth. First, in the third study, the intervention lasted 13 weeks, which was the longest duration among the FLSA studies. According to Spearman (1927) Law of Diminishing Returns (SLODR) (1927), it is reasonable to infer that as the intervention period progressed, the weekly intervention’s mitigating effect on learners’ FLA might diminish. In addition, results in the third study also recorded that most students held negative perceptions toward chatbot interaction. Thus, learners may also have developed resistance to the AI intervention due to anxious and awkward feelings, ultimately leading to an increase in FLA levels. Second, in the fifth study, participants generally exhibited varying degrees of anxiety prior to the intervention. The results of the fifth study indicated that, learners’ apprehension of how others (including teachers and peers) would evaluate their performance intensified after AI intervention, whose impact outweighed that of AI intervention, leading to an increase in FLA level. Finally, the fourth study was a one-shot intervention targeting FLRA whose intervention period was shorter than that of another study related to FLRA. Consequently, the results showed no significant changes. El Shazly (2021) interpreted this as “facilitative anxiety” and speculated that it might be related to the technical limitations of AI (situational factors) and learners’ high expectations (individual internal factors). These conflicting results suggest that the impact of AI on FLA may be moderated by variables such as intervention duration, technological maturity and learner characteristics, presenting a new direction for future research.

**TABLE 4:** AI effects on EFL learners FLA.

Effects on EFL learners FLA level		Number of studies
FLSA	Slightly intensified FLSA	2
	Reduced FLSA	13
FLRA	Significant reduced FLRA (with a small effect size)	1
	No significant change	1
FLWA	Reduced FLWA	1
FLLA	Decreased FLLA	1

## 4 Discussion

### 4.1 Considering the diversification of the research samples and the integrity of the study design

Based on the analysis of RQ1, researches on the impact of AI on EFL learners’ FLA have primarily focused on tertiary education and formal learning environments, with most researchers concentrating on intermediate-level learners and those from universities. Furthermore, mixed-methods research has emerged as a major trend in this realm. Although this approach combines the collection of both quantitative and qualitative data, issues regarding sample selection persist: Firstly, most studies have small sample sizes, short intervention periods, and lack cultural diversity. Additionally, most studies do not conduct long-term follow-up interviews or in-depth investigations with participants, raising questions about the reliability of the experimental results. These limitations may give rise to other issues:

First, most studies have focused on intermediate-level learners, neglecting the distinct characteristics of learners at other educational levels, which limits the generalizability and applicability of their conclusions (Ercikan and Roth, 2014). The conclusions drawn from these studies may not apply to learners in basic education (elementary, junior, and senior secondary), advanced learners, or other complex learner groups. Thus, AI’s impact across all educational tiers remains incompletely elucidated. The emphasis placed on learners at the tertiary education level also reflects a skewed allocation of educational resources. Some researchers, who may be affiliated with universities and educational institutions, tend to allocate research funding and effort toward these learners due to the convenience of conducting research and collecting data; this phenomenon further exacerbates the lag in access to the latest technologies and information among learners at other educational levels. Second, most studies are confined to formal educational settings and lack cultural and geographical diversity. This is likely due to the constraints of local educational systems and cultural mindsets, which may significantly limit the generalizability of conclusions to learners from different educational and cultural backgrounds; such conclusions may not be applicable to other contexts. Finally, most studies have small sample sizes and short intervention duration, which may limit the statistical significance (Aguirre-Urreta et al., 2024) and external validity (Creswell and Creswell, 2018) of the results. This may be due to limitations in the research environment and technology, preventing ideal research designs from being fully implemented in real-world settings.

The above statements indicate that research in this field needs to expand its research contexts and broaden its research perspectives. Future research requires close collaboration among AI technicians, education policymakers, researchers, teachers, and students to comprehensively consider teaching environments, educational facilities, and learner backgrounds, thereby enhancing the feasibility of research designs and the reliability of research findings. It is also vital to broaden the scope of research to include EFL learners from diverse cultural backgrounds, thereby improving the cultural and regional diversity of research conclusions. In addition, longitudinal studies after interventions should be further strengthened to ensure the stability of data and conclusions.

### 4.2 Introducing AI into EFL teaching and learning appropriately

RQ2 synthesizes the application of artificial intelligence technologies in EFL learners’ FLA from 2021 to 2025, specifically distinguishing among various technologies, including AI chatbots, generative LLMs, AI-driven tools, ASR systems and so on, which have been adopted in different pedagogical contexts. Included studies suggest AI holds considerable promise for mitigating EFL learners’ FLA. Diverse AI functionalities converge into three key affordances: providing immediate/real-time feedback, delivering personalized guidance, and enabling interactive practice. These affordances’ efficacy stems from their advantages in supporting language learning at cognitive and affective levels. Specifically, immediate feedback facilitates error correction and mastery, fostering intrinsic motivation and enhancing speaking self-efficacy (Shadiev et al., 2024; Zheng et al., 2025; Peng, 2025). Personalized guidance mitigates negative emotions, creates non-judgmental/low-pressure environments (reducing anxiety), and enhances learner autonomy (Wu et al., 2025; Xiao, 2025; Ebadi et al., 2025; Peng, 2025; Chen et al., 2025; Alsager, 2024). Interactive practice allows human-AI interaction, aiding strategic skill acquisition, improving oral fluency, and promoting holistic language development (Wu et al., 2025; Çelik et al., 2024), in other words, some AI technologies can act as a scaffolder (Vygotsky, 1978) to promote EFL learners’ certain language skill.

Nevertheless, the adoption of artificial intelligence (AI) technology to address the field of EFL learners’ FLA has also presented some theoretical and practical challenges, particularly regarding the transformation of the teacher’s role and learner autonomy. On the one hand, some researches have introduced AI into the teaching environment and contrasted it with traditional teacher-led instruction; as a result, the teacher’s role as a knowledge transmitter faces a significant shift, transforming into that of a guide for AI technology use and a facilitator of student-centered learning. Consequently, this suggests that teachers need to further explore new teaching methods and approaches to accommodate to future models of AI-integrated education. On the other hand, learners have shown a greater willingness to use AI tools to support their learning. Since AI can create a non-judgmental environment and alleviate learners’ fear and tension when interacting with others, thus alleviate FLA, ultimately boosting learner autonomy. However, attention must also be paid to the potential issue of learners developing a dependency on AI in the long term.

### 4.3 Exploring AI possible mechanisms or pathways on FLA

As analyzed in RQ3 regarding the mechanism by which AI varies EFL learners’ FLA levels, two possible pathways, the direct pathway and the indirect pathway, were proposed based on Fishbein and Ajzen’s (1975) Cognition-Affect-Conation Pattern (CAC) model. Among the 21 selected studies, most of them did not measure single FLA data, they also measured other related variables simultaneously. Many studies mentioned Foreign Language Enjoyment (FLE) as one of emotional experiences that may also be a factor leading to changes in FLA. However, it should be noted that most current studies focus on the main effects of AI interventions, with few studies conducting statistical tests on mediating or moderating mechanisms. Future research could conduct correlation analyses between the measured variables and FLA to clarify the interdependent relationships among them. This would help explore other possible potential mechanisms profoundly through which AI exerts influences FLA, provide multiple feasible approaches to mitigate FLA in the field of AI-assisted foreign language education, and ultimately enhance learners’ foreign language skill.

Furthermore, as indicated by the CAC model, “affection” occupies a central position, serving as a bridge connecting “cognition” and “conation.” In this systematic review, FLA, as one manifestation of emotion, can reasonably be presumed to play a key role in potential pathways of AI impacts. As one of the outcomes of AI intervention, FLA itself may serve as a mediator or a moderator influencing other variables, or it may be held sway over by other factors. Most current studies focus on changes in FLA at the end of the intervention, while few examine the role it may play throughout the entire AI intervention process.

Currently, in the fields of AI-assisted language learning and educational psychology, only a few of existing research has attempted to elucidate the mechanisms through which AI influences FLA from various theoretical perspectives. In the field of foreign language learning, anxiety has long been a major area of research (MacIntyre, 2017). Existing studies drawing on Cultural-Historical Activity Theory (CHAT) (Choi, 2025) and Activity Theory (Ebadi et al., 2025) have both acknowledged the role of AI in this process. The former views AI as an element capable of reshaping the entire learning activity system, while the latter posits that learners can access their Zone of Proximal Development (ZPD) through collaborative or interactive activities with AI. Additionally, some studies have explored FLA and related positive psychological constructs (such as FLE) from the perspective of Positive Psychology (Zhang et al., 2024; Wu et al., 2025). However, these studies have focused only on specific AI tools or specific types of FLA. Since studies on the impact of AI on EFL learners’ FLA is in a rapidly developing but still nascent stage, no study has yet provided a comprehensive analysis of this topic. Thus, summarizing the effects of various types of AI on different forms of FLA, this study adopts the CAC model as the framework to explain the possible mechanism of AI in EFL learners’ FLA, offering a new perspective for FLA researches. This theoretical perspective synthesizes relatively scattered findings into a systematic, dynamic, and more explanatory framework, attempting to clarify whether AI may ultimately influence learners’ behavior by altering their cognition or their emotions. Two proposed possible pathways also help elucidate the potential mechanisms and complexity of AI’s impacts on FLA, which may extend existing researches in this field.

### 4.4 Paying heed to AI limitations and learners’ voices

RQ4 reviews the potential factors in the selected studies that may carry wight with the role of AI in FLA, categorizing them primarily into internal and external factors. Internal factors concentrate primarily on the learners themselves, that is, the individual differences among learners and their perceptions on AI application, which may bring impacts to AI’s effectiveness on FLA. Prior proficiency, digital literacy, and learning style preferences may also cause effects on learners’ perceptions toward AI tools (Jeon, 2024). Therefore, clarifying these different learner characteristics will deepen the understanding of how to select appropriate AI chatbots based on learner needs to alleviate FLA. It is worth noting that while the qualitative data from most studies indicate that learners hold positive impressions of AI, the random selection of interview participants and the limited number of interviews may have prevented theoretical saturation (Glaser and Strauss, 2017), potentially leading to an overestimation of AI’s impacts on FLA. Furthermore, when introducing such technologies in the classroom, teachers should consider students’ perspectives or genuine needs and implement this intervention with caution.

External factors take into account the inherent limitations of AI and the learning context. AI itself has inherent limitations. Some studies went beyond simply introducing AI into traditional teaching and instead integrated it into teaching innovation (Li and Long, 2025) and classroom activities (Wu et al., 2025). It remains to be seen whether these methods, compared to traditional teaching models, might have a positive effect on FLA to some extent. Qualitative data from some studies mentioned learners’ perspectives on AI application, implicitly involving factors related to learner interactions between peers and teachers, which may also have a bearing on AI effectiveness on FLA. Therefore, it is essential to strike a balance between the adoption of AI and teacher-learner interactions. Future research could explore the change of FLA under the condition that compares the differences between peer-learner interaction and AI-learner interaction, thereby further elucidating the issue of AI’s substitutability as an interaction partner. Additionally, the inherent limitations of AI itself may also be instrumental in research outcomes. During human-chatbot interactions, the information provided by AI may be difficult for learners to understand due to a lack of conciseness or clarity; LLMs may cause learners to experience anxiety about being unable to communicate through body language due to digressions from the topic; and internet connectivity issues may also affect the processing of AI-generated information. Therefore, during the intervention process, researchers and AI practitioners must consider how to mitigate the interference caused by these factors, and resolve these issues will enhance the reliability of research findings.

## 5 Limitations

This study presents several limitations. First, the scope of the reviewed literature is defined by specific inclusion criteria and restricted to studies published in English. Consequently, the exclusion of non-English research may have led to the omission of valuable insights and emerging perspectives of EFL researches from other linguistic contexts, which may lead to the generalizability of the findings. For example, among the included studies, nearly half of them involved Chinese EFL learners. Given the significant differences among countries or regions in their educational systems and institutions, classroom practices, teaching technology infrastructure, and learners’ attitudes toward AI, the conclusions drawn primarily from Chinese context may not be directly adapted to other countries or regions with different sociocultural and educational backgrounds. Second, due to the high quality and data stability of peer-reviewed journals, the exclusion of other formats, such as conference papers, book chapters, imposes domain-specific cognitive limitations on our findings. In light of the rapid development of AI, our reliance on peer-reviewed journals has resulted in a significant technological lag, that is, the included studies may fail to reflect the potential impact of cutting-edge AI on FLA. Consequently, our systematic review may tend to underestimate the potential role of contemporary AI in alleviating FLA. Third, the study specifically investigates the impact of artificial intelligence (AI) on English as a Foreign Language (EFL) learners’ Foreign Language Anxiety (FLA), an emerging field where scholarly activity is growing but the published literature remains relatively limited. Consequently, the small number of identified studies meeting the inclusion criteria restricts the breadth and generalizability of the findings. Fourth, the article screening process places excessive emphasis on high-quality journals and methodological rigor, which may have led to the neglect of innovative practices and emerging trends in this field. Fifth, this paper does not specify the reviewer agreement in the screening and coding process, even though the reviewers ultimately reached a consensus. Future research should include this data to enhance the transparency of the screening and coding process. Finally, although this systematic review adheres strictly to PRISMA guidelines and employed rigorous screening procedures for the included 21 studies, the modest sample size poses a constraint for robust quantitative analysis and increases the potential risk of publication bias. Consequently, this review has focused predominantly on a qualitative analysis of the selected studies; comprehensive quantitative synthesis might become feasible as the volume of published work in this area expands.

Furthermore, it is important to acknowledge that existing research in this field also exhibits limitations. First, the limited sample sizes and context-specific constraints observed in several primary studies restrict the generalizability of their individual findings. Second, most existing studies lack mediation analyses concerning the variables associated with AI’s application in mitigating EFL learners’ FLA. Instead, these studies primarily analyze and report data on involved variables individually, thereby failing to elucidate the potential mediating pathways through which AI interventions might leave a mark on FLA. Third, the effectiveness of AI in EFL education is likely impacted by factors extending beyond purely technological considerations, such as pedagogical approaches, learner acceptance, and institutional support. Future research should therefore investigate the complex factors impacting the implementation and effectiveness of AI in EFL contexts, insights which would significantly advance its optimal application within educational settings.

## 6 Conclusion

This study presents a systematic review of 21 empirical studies examining the impact of artificial intelligence on English as a Foreign Language learners’ Foreign Language Anxiety. The primary contributions of this work are summarized as follows. First, the review categorizes AI technologies applied in EFL contexts and classifies their effects on learner outcomes according to cognitive, affective and conative dimensions, thereby offering a structured overview of AI’s multifaceted influence. Second, based on CAC model, the study interprets the possible mechanism of AI’s impacts on EFL learners’ FLA with a two-pathway effect, synthesizing the primary findings of learners’ changes from selected researches. The proposed potential mechanisms have not yet been empirically validated and may serve as a direction for future research in this field. Third, the analysis identified potential mediating or moderating pathways implicitly suggested by some reviewed researches, such as AI interventions reducing anxiety potentially through influencing specific affective responses. Future empirical studies are needed to explicitly test these posited mediation effects, addressing a critical gap in the current understanding. Fourth, the study highlights key factors that potentially moderate the impact of AI on EFL learners’ FLA and influence its adoption and practical effectiveness in education, providing valuable focal points for subsequent research. The findings of this review are expected to contribute significant novel insights to the existing knowledge base on AI-assisted EFL instruction. They offer valuable guidance for researchers, educators, and policymakers seeking to understand and harness the potential of AI in EFL pedagogy, particularly through a mediating perspective.

## Data Availability

The original contributions presented in the study are included in the article/Supplementary material, further inquiries can be directed to the corresponding authors.
