# Supplementary material for: Research on the impact of artificial intelligence on EFL learners’ foreign language anxiety
Source: Front Psychol. 2026 Aug 26;17:1847269. doi: 10.3389/fpsyg.2026.1847269 (PMC13553964; doi:10.3389/fpsyg.2026.1847269)
Supplement: Supplementary file 1 [file Supplementary_file_1.docx]

**Appendix 1**

Authors, AI technologies, FLA types, participants, study settings, study designs, and intervention duration.

| **No.** | **Author (Year)** | **AI technology** | **FLA type** | **Participants** | **Study setting** | **Study design** | **Intervention duration** |
| --- | --- | --- | --- | --- | --- | --- | --- |
| 1 | Li & Long (2025) | LLM (ChatGPT and DeepSeek) | FLSA | 150 undergraduate students from a key provincial university in China | Formal learning | Mixed | 6-week comparative experiment |
| 2 | Zhang et al. (2024) | AI-driven tool (Lora app) | FLSA | 131 Chinese university students within the CEFR levels of B2 and C1 (Freshman English majors), 65 in EG (female=47; male=18),66 in CG (female=52; male=14) | Informal learning | Quantitative | 6-week of session |
| 3 | Çakmak (2022) | AI chatbot (Replika) | FLSA | 90 Turkish university students (Freshman English Language Teaching majors) (female=57; male=33) | Formal learning | Mixed | 13-week of task |
| 4 | Çelik et al. (2024) | LLM (ChatGPT) | FLRA | 105 Turkish university students at the CEFR level of B1(Prep school students) (female=60; male=45) | Formal learning | Quantitative | 20-min of one shot intervention |

Cont.

| **No.** | **Author (Year)** | **AI technology** | **FLA type** | **Participants** | **Study setting** | **Study design** | **Intervention duration** |
| --- | --- | --- | --- | --- | --- | --- | --- |
| 5 | El Shazly (2021) | AI chatbot (Audrey, Charles, Cristal, and Mike; Mondly) | FLSA | 48 Egyptian university freshman students at the CEFR level of B1/B2(mean age = 18.9), (female=38; male=10) | Formal learning | Mixed | 6-week of course |
| 6 | Wu et al. (2025) | AI chatbot (Replika) | FLSA | 75 Freshman English majors Indonesian university students 39 in EG(female=36; male=3), 36 in CG(female=27; male=9) | Formal learning | Mixed | 4-week of course |
| 7 | Shadiev et al. (2024) | ASR system (SECF technology) | FLSA | 28 university students from China (n=14) and Kazakhstan (n=14) | Formal learning | Mixed | 10-week of learning activity |
| 8 | Chen (2024) | AI-driven tool (Orai, Virtualspeech) | FLSA | 33 Taiwanese university students (female=26; male=7), 30 from the College of Social Science, and 3 from the College of Business, at the CEFR level of A2 to B1, 11 in CG, 11 in EG1, 11 in EG2 | Formal learning | Mixed | 3-week of course |

Cont.

| **No.** | **Author (Year)** | **AI technology** | **FLA type** | **Participants** | **Study setting** | **Study design** | **Intervention duration** |
| --- | --- | --- | --- | --- | --- | --- | --- |
| 9 | Dizon & Gold (2023) | AWE system (Grammarly) | FLWA | 58 Japanese university students, ranged from 18 to 20 years old, at the CEFR level of B1 to B2 (female=36; male=22) | Formal learning | Mixed | 16-week of course |
| 10 | Ebadi et al. (2025) | AI-driven tool (SmallTalk2Me) | FLSA | 64 Iranian university students (IELTS prep course) at the CEFR level of B2(female=39; male=25) 32 in EG, 32 in CG | Formal learning | Mixed | 6-week of course |
| 11 | Chen et al. (2022) | ASR system (a dynamic assessment-based speech recognition (DA-SR) system) | FLSA | 56 Taiwanese elementary school students of 5th grade (10- or 11-year-old),  30 in EG, 26 in CG | Formal learning | Quantitative | 3-week of course |
| 12 | Ding & Yusof (2025) | AI chatbot (Mondly) | FLSA | 60 Chinese university students taking IELTS prep course at the CEFR level of B1 (female=36; male=24) 30 in EG, 30 in CG | Formal learning | Mixed | 6-week of session |
| 13 | Peng (2025) | LLM (ChatGPT) | FLA | 30 Chinese EFL students (college students) from four universities | Formal learning | Qualitative | 28 days |

Cont.

| **No.** | **Author (Year)** | **AI technology** | **FLA type** | **Participants** | **Study setting** | **Study design** | **Intervention duration** |
| --- | --- | --- | --- | --- | --- | --- | --- |
| 14 | Zheng (2024) | AI chatbot (Reading Bot) | FLRA | 84 Chinese junior high school students of 7th grade, (11 to 14 years old)(female=46; male=38) 42 in EG, 42 in CG | Formal learning | Mixed | 45mins×5(sessions) |
| 15 | Xiao (2025) | AI-driven tool (Google text-to-speech (TTS) app) | FLLA | 84 EFL Chinese university students (female=48; male=36) 42 in EG, 42 in CG | Formal learning | Quantitative | 8-week of session |
| 16 | Wang et al. (2024) | AI chatbot (Typebot, D-ID agent) | FLSA | 99 Chinese university students (from a language institution)at the CEFR level of B1 to B2, 33 in CG, 33 in EG1, 33 in EG2 | Formal learning | Mixed | 6-week of course |
| 17 | Zheng et al. (2025) | LLM (GPT-4) | FLSA | 83 Chinese university freshmen students (female=57; male=26) 41 in EG, 42 in CG | Formal learning | Mixed | 4-week of session |
| 18 | Wang & Wen (2025) | AI-driven tool (Doubao) | FLSA | 147 Chinese EFL learners (female=80; male=67) | Formal learning | Quantitative | 20mins×10(sessions) |
| 19 | Choi (2025) | LLM (ChatGPT-4o) | FLSA | 15 high school EFL students at the CEFR level of B2 to C1 | Informal learning | Mixed | 8-week of session |

Cont.

| **No.** | **Author (Year)** | **AI technology** | **FLA type** | **Participants** | **Study setting** | **Study design** | **Intervention duration** |
| --- | --- | --- | --- | --- | --- | --- | --- |
| 20 | Chen et al. (2025) | LLM (ChatGPT-4o) | FLSA | 71 Chinese EFL university students:  the high proficiency group (HG, n = 37) and the low proficiency group (LG, n = 34) | Formal learning | Mixed | 6-week of task |
| 21 | Alsager (2024) | LLM (ChatGPT) | FLA | 83 Saudi Arabian university students (English majors, aged 21-26), 41 in EG, 42 in CG | Formal learning | Quantitative | 16 sessions |
